# Surgical resection following chemoradiotherapy for thoracic SMARCA4-deficient undifferentiated tumor: a report of two cases

**DOI:** 10.1186/s40792-024-02053-y

**Published:** 2024-11-05

**Authors:** Kensuke Takei, Mitsuhiro Isaka, Junji Wasa, Takuya Kawata, Tatsuya Masuda, Shinya Katsumata, Koki Maeda, Hideaki Kojima, Hayato Konno, Yasuhisa Ohde

**Affiliations:** 1 Division of Thoracic Surgery, Shizuoka Cancer Center, Shimonagakubo 1007, Nagaizumi-cho, Sunto-gun, Shizuoka, 411-8777 Japan; 2 Division of Orthopedic Oncology, Shizuoka Cancer Center, Shizuoka, Japan; 3 Division of Pathology, Shizuoka Cancer Center, Shizuoka, Japan

**Keywords:** SMARCA4-deficient undifferentiated tumor, Thoracic neoplasm, Thoracic surgery, Chemoradiotherapy

## Abstract

**Background:**

Thoracic SMARCA4-deficient undifferentiated tumor (SMARCA4-UT) is a high-grade malignant neoplasm with a poor prognosis. Most cases of SMARCA4-UT have extensive chest wall and mediastinum involvement. The efficacy of surgical resection has not been clearly established. Here, we report two surgical cases of SMARCA4-UT with chest wall invasion after chemoradiotherapy.

**Case presentation:**

The first patient was a 40-year-old man with back pain. Computed tomography revealed a 6.8 cm mass in contact with the thoracic vertebrae near the intervertebral foramen, which was suspected to involve the third to fifth ribs. The patient was diagnosed with SMARCA4-UT with clinical T3N0M0 stage IIB. The tumor shrank after chemoradiotherapy, and conversion surgery combined with partial vertebrectomy was performed. Histopathological findings revealed 30% residual tumor in the tumor bed. Thirty-six days after surgery, the patient developed multiple liver metastases and peritoneal dissemination. Chemotherapy combined with immune checkpoint inhibitor treatment was performed, resulting in tumor shrinkage. However, peritoneal dissemination recurred within a short interval. The patient died 5 months postoperatively. The second patient was a 74-year-old man with chest pain. Computed tomography revealed a 7.4-cm mass in the left upper lobe with invasion of the third and fourth ribs. The patient was initially diagnosed with non-small cell lung cancer with clinical T4N1M0 stage IIIA. The tumor shrank after induction chemoradiotherapy, and a left upper lobectomy combined with the chest wall resection was performed. Based on histopathological findings, the patient was diagnosed with SMARCA4-UT. The residual tumor percentage was 3%. The patient was followed up for 12 months postoperatively without recurrence.

**Conclusions:**

We performed the complete resection of SMARCA4-UT following chemoradiotherapy. The two surgical cases had different postoperative courses. Radical surgery after chemoradiotherapy is effective for local control. However, its long-term prognostic efficacy remains unclear. Multidisciplinary approaches and further investigations of novel therapeutic options are required.

## Background

Most cases of thoracic SMARCA4-deficient undifferentiated tumors (SMARCA4-UT) are found at advanced stages and have extensive involvement in thoracic structures, including the chest wall and mediastinum [1]. The outcomes of surgery and radiotherapy are unclear [2, 3]. Here, we present two cases of SMARCA4-UT with chest wall invasion that underwent complete resection after chemoradiotherapy (CRT).

## Case presentation

### Case 1

**Fig. 1:**
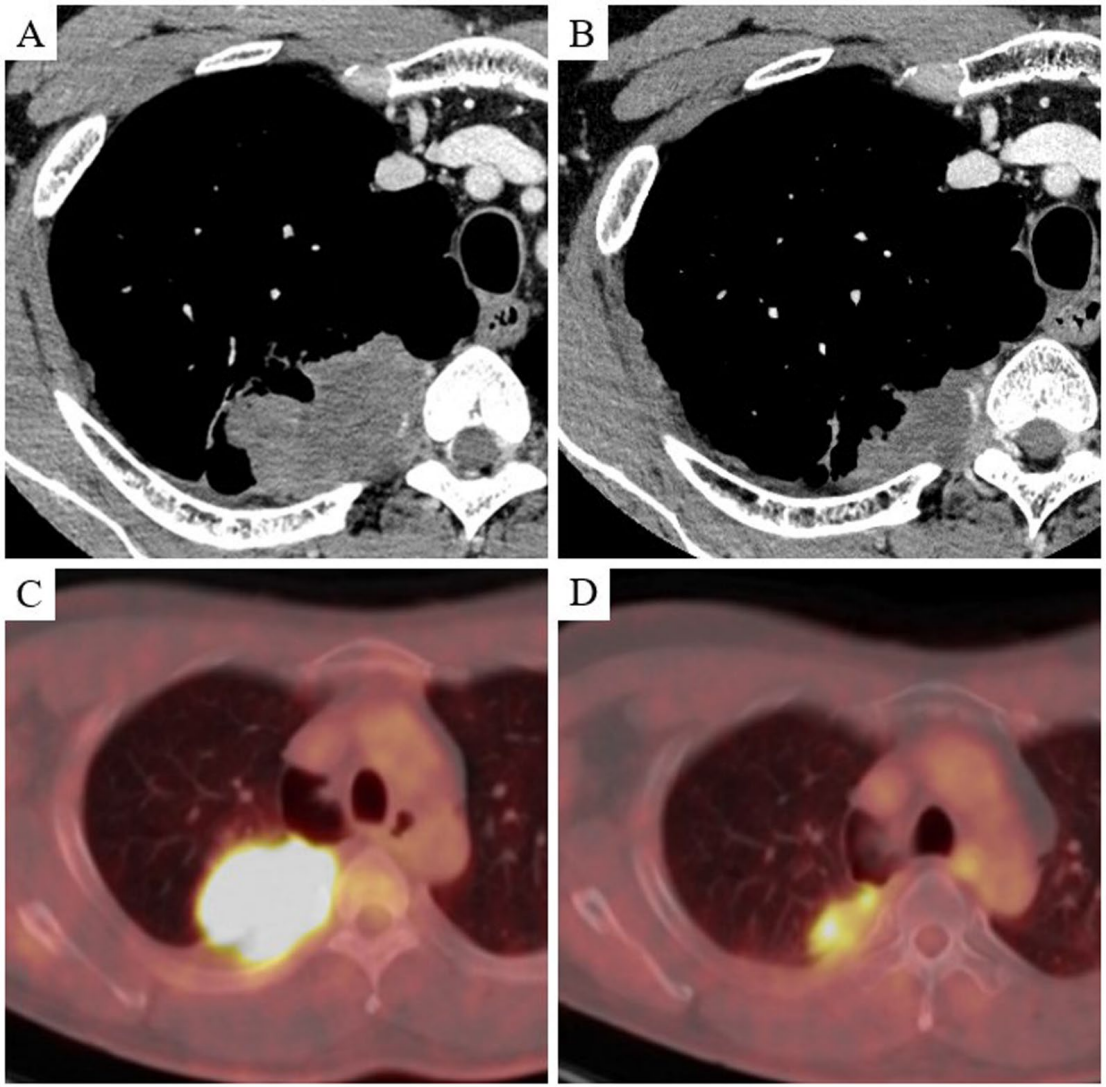
Computed tomography (CT) and 18F-fluorodeoxyglucose positron emission tomography (FDG-PET)/CT images of case 1. **A** Contrast-enhanced CT showing a 6.8-cm mass with chest wall invasion, including the fourth rib, in contact with the intervertebral foramen. **B** CT showed that the tumor shrank to a maximum diameter of 4.5 cm following chemoradiotherapy. **C** The maximum standardized uptake value (SUVmax) of the tumor on FDG-PET/CT was 12.9 before chemoradiotherapy. **D** The SUVmax of the tumor decreased to 6.1 after chemoradiotherapy

A 40-year-old man presented with back pain. He had smoked two packs of cigarettes per day for 25 years. The patient had no comorbidities. Chest computed tomography (CT) revealed a mass measuring 6.8 cm in diameter (Fig. 1A). The mass was suspected to involve the third, fourth, and fifth ribs and was in contact with the thoracic vertebrae near the intervertebral foramen. Enhanced brain magnetic resonance imaging and 18F-fluorodeoxyglucose positron emission tomography (FDG-PET)/CT revealed no lymph nodes or distant metastases. The specimen obtained by CT-guided biopsy showed the proliferation of homogeneous tumor cells with weak cohesiveness and a complete loss of SMARCA4 expression on immunohistochemical examination. The patient was diagnosed with SMARCA4-UT with clinical T3N0M0 stage IIB. Complete resection by initial surgery was difficult in terms of tumor margins around the intervertebral foramen, and assessment was planned following definitive CRT (cisplatin, 80 mg/m^2^; vinorelbine, 20 mg/m^2^; and intensity-modulated radiotherapy (IMRT)). After two cycles of chemotherapy and a total radiation dose of 50 Gy, the tumor shrank to a maximum diameter of 4.5 cm (Fig. 1B). The treatment effect was classified as a partial response, according to the Response Evaluation Criteria in Solid Tumors version 1.1. The maximum standardized uptake value (SUVmax) of the tumor on FDG-PET/CT decreased from 12.9 to 6.1 before and after chemoradiotherapy (Fig. 1C, D).

**Fig. 2:**
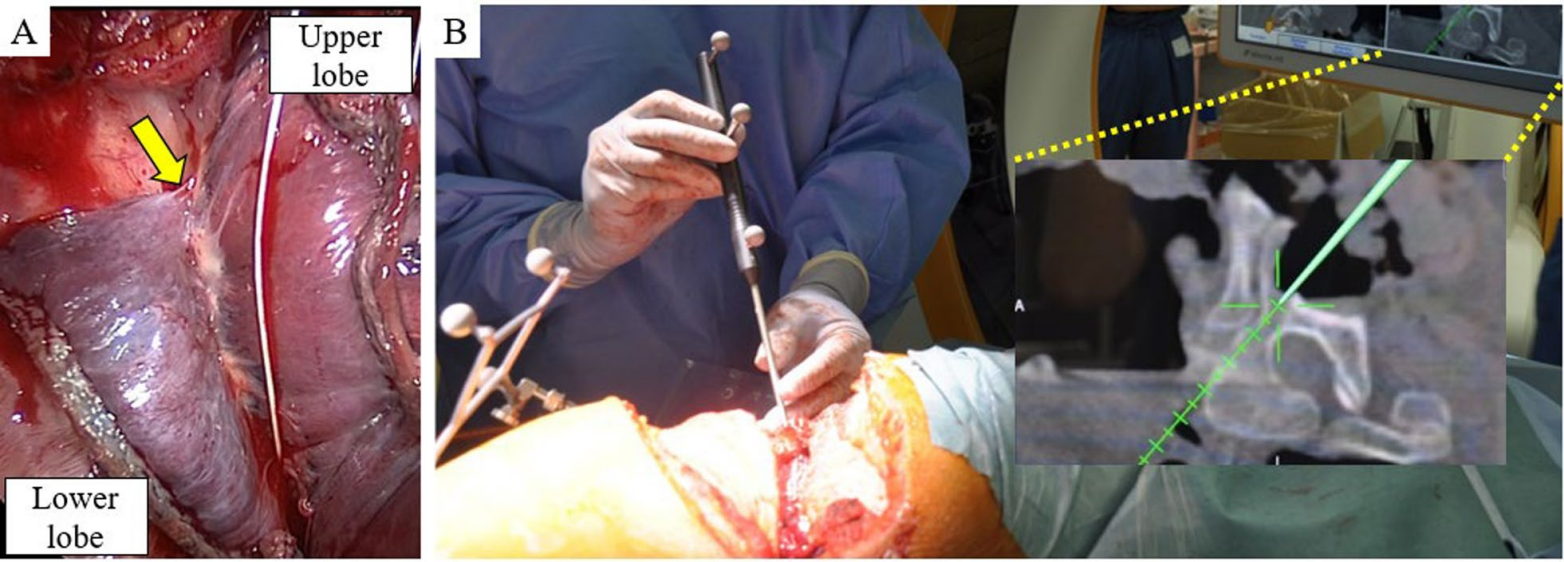
Intraoperative findings in case 1. **A** Intraoperative findings showed that the tumor was primarily located in the chest wall and involved both upper and lower lobes (arrow). **B** A CT-based navigation system was used for vertebrectomy, and the monitor shows the planned cutting line in green

**Fig. 3:**
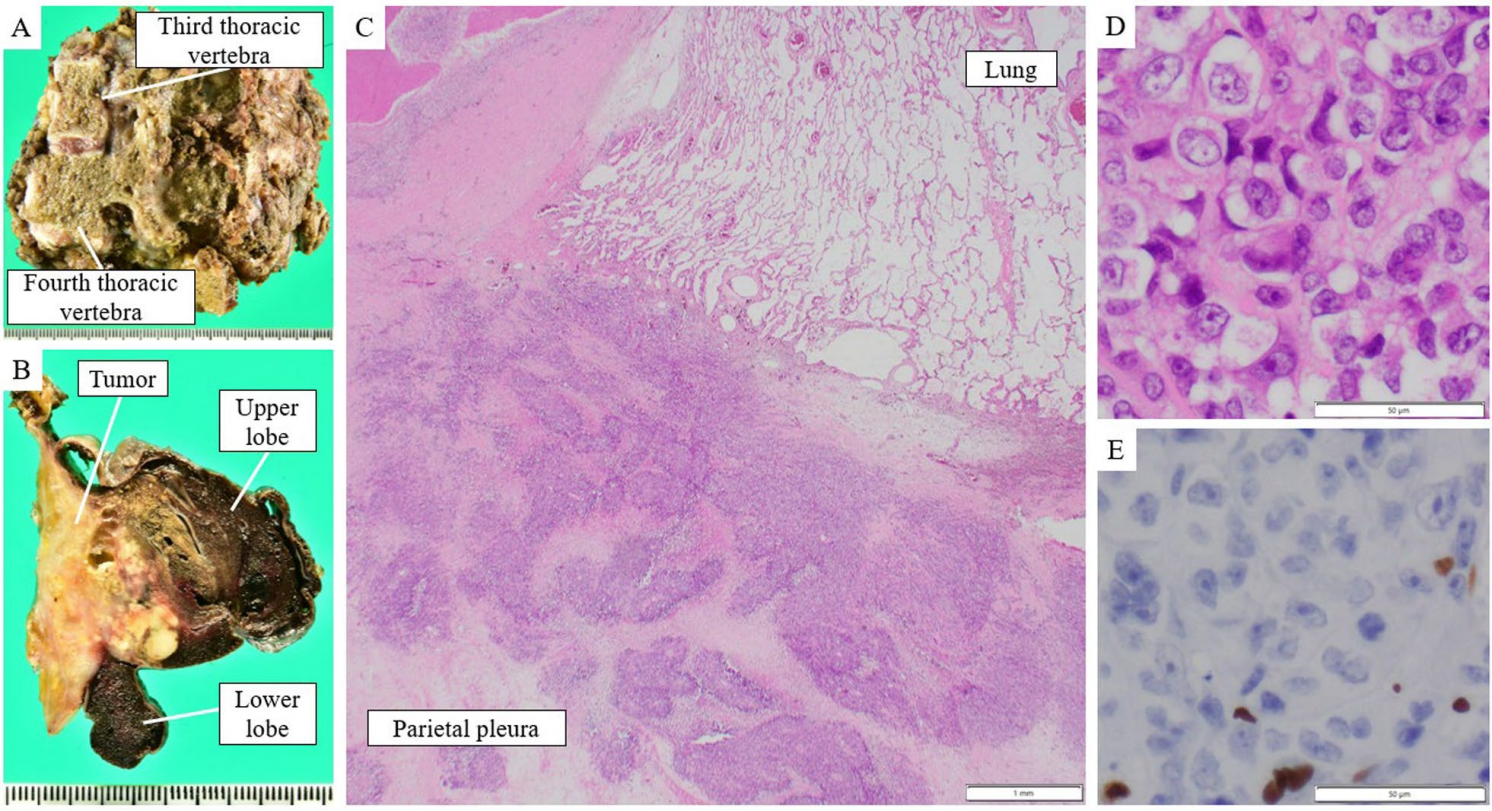
Pathological findings of case 1. **A** Macroscopic findings showing that the tumor was resected en bloc with the ribs and thoracic vertebrae. **B** Cut surface showing that the tumor was primarily located in the chest wall. **C**, **D** Hematoxylin and eosin-stained images show that the tumor was composed of sheets of large tumor cells without keratinization or glandular or rosette formation. No viable tumor cells were found in the lung parenchyma (scale bar, **C** 1 mm, **D** 50 µm). **E** Immunohistochemical features indicate the loss of SMARCA4 (scale bar, 50 µm)

In contrast, the tumor area around the intervertebral foramen remained unchanged. We decided to perform conversion surgery because additional radiotherapy to the peri-spine was challenging owing to adverse effects on the spinal cord. The patient was placed in a left lateral decubitus position. After confirming that there was no dissemination or malignant pleural effusion via thoracoscopy, we initiated surgery through a large posterolateral incision, known as the Paulson approach. Surgical findings revealed that the tumor was primarily located in the chest wall, with minor infiltration into the margins of the upper and lower lobes (Fig. 2A). Therefore, the primary site of the tumor was determined to be the chest wall, and wedge resection of the right upper and lower lobes combined with resection of the third, fourth, and fifth ribs was initially performed. A CT-based navigation system (Brainlab AG, Munich, Germany) was used to determine the vertebral cutting lines with a margin from the tumor (Fig. 2B). Subsequent partial vertebrectomies were performed without prone positioning, while observing the thoracic cavity and posterior aspect of the vertebral body viewed in the same surgical field, and the tumor was resected en bloc (Fig. 3A). The cut surfaces of the macroscopic specimens revealed that the tumor was primarily located in the chest wall (Fig. 3B). Based on histopathological findings, the tumor was composed of sheets of large tumor cells without keratinization or glandular or rosette formation. The tumor bed was mainly located in the chest wall, and viable tumor cells were observed in the parietal pleura and periosteum of the ribs but not in the lung parenchyma. The percentage of residual tumors in the tumor bed was approximately 30% (Fig. 3C, D). The surgical margins were negative, and complete pathological resection was achieved. Immunohistochemical examination revealed a complete loss of SMARCA4 expression (Fig. 3E). Other immunohistochemical features indicated the expression of CD34 and a lack of TTF-1, p40, INSM1, and SALL4. The programmed death ligand-1 (PD-L1) tumor proportion score was 1%. The patient developed abdominal distention and tenderness on postoperative day 36, and a whole CT revealed multiple liver metastases, peritoneal dissemination, and ascites. Four courses of atezolizumab, bevacizumab, paclitaxel, and carboplatin were administered, resulting in tumor shrinkage and disappearance of ascites. However, peritoneal dissemination recurred within a short period of one month, and second-line treatment was not possible owing to Performance Status. Five months after surgery, the patient died.

### Case 2

**Fig. 4:**
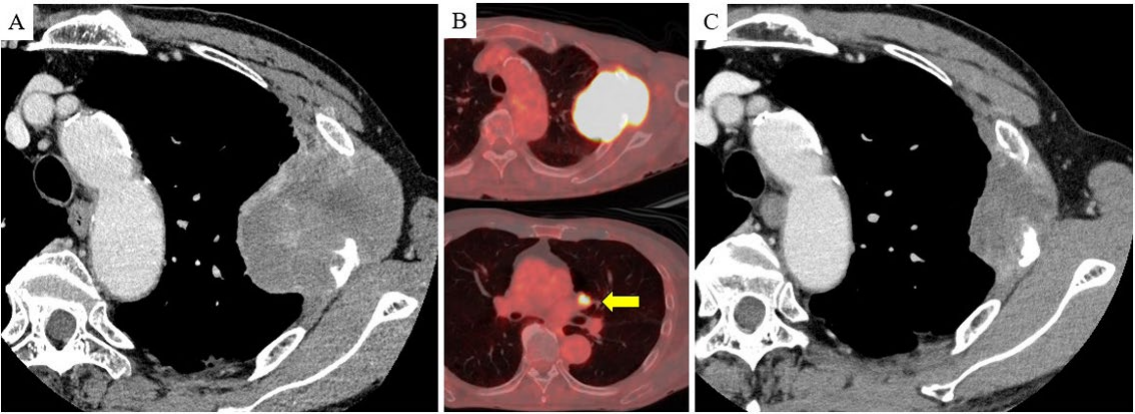
Computed tomography (CT) and 18F-fluorodeoxyglucose positron emission tomography (FDG-PET)/CT images of case 2. **A** Contrast-enhanced CT showing a 7.4-cm mass with chest wall invasion, including the third and fourth ribs. **B** FDG-PET/CT showed hilar lymph node metastasis (arrow). The maximum standardized uptake value (SUVmax) of the primary tumor was 15.4. **C** CT showed that the tumor shrank to a maximum diameter of 4.7 cm following chemoradiotherapy

**Fig. 5:**
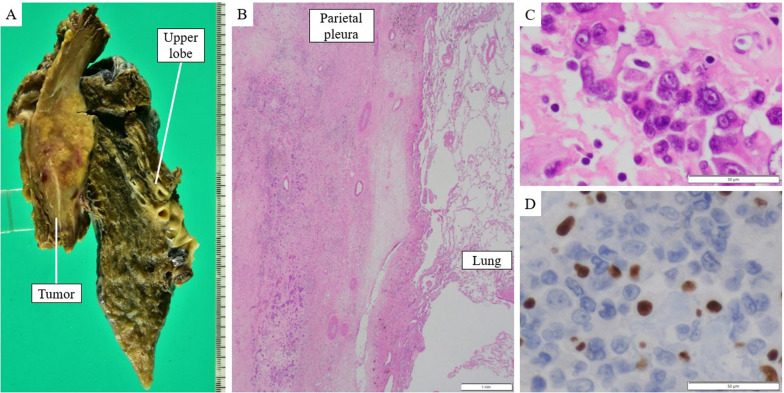
Pathological findings of case 2. **A** Surface of the macroscopic specimen, showing that the tumor was primarily located in the chest wall. **B**, **C** Hematoxylin and eosin-stained images show that the tumor cells have prominent nucleoli without keratinization or glandular or rosette formation. No viable tumor cells were found in the lung parenchyma (scale bar, **B** 1 mm, **C** 50 µm). **D** Immunohistochemical features indicate the loss of SMARCA4 (scale bar, 50 µm)

A 74-year-old man presented with chest pain. He had smoked one pack of cigarettes per day for 54 years. He had hypertension and mild renal dysfunction but no other comorbidities. A CT showed a mass measuring 7.4 cm in diameter in the left upper lobe with invasion of the third and fourth ribs (Fig. 4A). FDG-PET/CT revealed hilar lymph node metastasis, and no distant metastases were observed. The SUVmax of the primary tumor was 15.4 (Fig. 4B). The specimen obtained by CT-guided biopsy showed a poorly differentiated carcinoma. The patient was diagnosed with non-small cell lung cancer (NSCLC) with clinical T4N1M0 stage IIIA. Radical surgery after CRT induction (carboplatin at an area under the concentration–time curve of 2 mg/mL/min, paclitaxel at 40 mg/m^2^, and IMRT) was planned. Chemotherapy was administered weekly for five cycles and terminated when the total radiation dose reached 50 Gy. The tumor shrank to a maximum diameter of 4.7 cm (Fig. 4C), and a partial response was achieved. Left upper lobectomy with mediastinal lymph node dissection combined with resection of the second to fifth ribs was performed. The cut surfaces of the macroscopic specimens showed that the tumor was primarily located in the chest wall (Fig. 5A). Based on the histopathological findings, the tumor cells showed prominent nucleoli without keratinization or glandular or rosette formation. The tumor bed was mainly located in the chest wall, and no viable tumor cells were observed in the lung parenchyma. The residual tumor percentage in the tumor bed was approximately 3% (Fig. 5B, C). Complete loss of SMARCA4 expression was observed (Fig. 5D). Other immunohistochemical features indicated SALL4 expression and a lack of TTF-1, p40, INSM1, and CD34. The PD-L1 tumor proportion score was 0%. After surgery, the patient was followed up for 12 months without adjuvant chemotherapy, and no recurrence was observed.

## Discussion

SMARCA4-UT was initially classified as a subtype of sarcoma [1, 4]. It is considered primarily to be smoking-associated undifferentiated carcinomas rather than primary thoracic sarcomas [1]. SMARCA4-UT presents as a large comprehensive mass in the mediastinum, pulmonary hilum, pleura, and lung [5]. There have also been reported cases in which the primary tumor site was the chest wall [4]. Immunohistochemically, complete loss of SMARCA4 expression is typical, and stem cell markers, including SALL4, CD34, and SOX2 are expressed in many cases [4, 5].

The prognosis of SMARCA4-UT is poor, with a median overall survival of 4–7 months [1, 2, 4, 6]. Most patients were treated with chemotherapy alone or palliation. The regimens used to treat sarcomas were not highly effective [2]. Cytotoxic chemotherapy alone was not considered to be effective for SMARCA4-UT. Standard treatment has not yet been established, and the therapeutic approach is in accordance with that used for NSCLC. However, surgical outcomes have not been well-reported. In a previous surgical case series, the median overall survival was 15.6 months [3]. There are few reports on radiotherapy for patients with advanced-stage disease with a poor prognosis [2].

In our cases, CRT with regimens used for NSCLC was effective, resulting in significant tumor shrinkage and complete resection combined with the chest wall. Combined vertebrectomy with tumor margins were safely performed using a CT-based navigation system while viewing the cutting line. Additionally, a large posterolateral incision, the Paulson approach, allows surgery to be performed from a single field of view without the prone position. Surgical resection following CRT may be effective for SMARCA4-UT in terms of local control.

Based on the histopathological findings in both cases, the tumor bed was primarily located in the chest wall. Viable tumor cells were observed only in the chest wall but not in the lung parenchyma. Therefore, the primary site of the tumor was considered to be the chest wall.

These two surgical cases had different postoperative courses. The relapse-free patient showed a major pathological response. In general, complete resection and major pathological responses result in a favorable prognosis for resected lung cancer [7]. The other patient, with 30% residual tumor in the tumor bed, had an early relapse with distant metastases. The development of effective adjuvant therapy is warranted to prevent distant metastatic recurrence in operable cases of SMARCA4-UT with many residual viable cells.

The efficacy of immune checkpoint inhibitors (ICIs) for SMARCA4-UT has been reported [8]. SMARCA4-UT has a high tumor mutation burden [1], which supports the potential efficacy of ICIs. PD-L1 expression, a biomarker for ICI treatment in NSCLC, shows variation in SMARCA4-UT, and its assessment is controversial [5]. In the genomic profile, SMARCA4-UT has a relatively high prevalence of *STK11* or *KEAP1* co-mutations after the *TP53* mutation [1]. Mutations in *STK11* and *KEAP1* are associated with resistance to ICI treatment in lung adenocarcinoma [9], but these mutations are difficult to sufficiently predict the efficacy of ICI treatment in SMARCA4-UT [5]. ICI plus platinum-based chemotherapy has been suggested to be effective in the advanced stages [10]. In the perioperative period, there has been a report of successful conversion surgery following chemotherapy combined with ICI [11]. Several clinical trials on ICI treatment for SMARCA4-deficient tumors are ongoing [5]. Perioperative ICIs may be key drugs for SMARCA4-UT to improve the prognosis.

## Conclusions

We performed the complete resection of SMARCA4-UT following CRT in two cases. Radical surgery after CRT is effective for local control. However, its long-term efficacy remains unclear. Furthermore, information on management strategies preventing distant metastatic recurrence is insufficient. Multidisciplinary approaches and the development of novel therapeutic options are required.

## Data Availability

The data supporting the conclusions of this article are included within the article.

## Abbreviations

SMARCA4-UT: SMARCA4-deficient undifferentiated tumor
CRT: Chemoradiotherapy
CT: Computed tomography
FDG-PET: 18F-fluorodeoxyglucose positron emission tomography
IMRT: Intensity-modulated radiotherapy
SUVmax: Maximum standardized uptake value
PD-L1: Programmed death ligand-1
NSCLC: Non-small cell lung cancer
ICI: Immune checkpoint inhibitor
